# Supplementary material for: Multi‐dataset identification of innovative feature genes and molecular mechanisms in keratoconus
Source: J Cell Mol Med. 2024 Sep 19;28(18):e70079. doi: 10.1111/jcmm.70079 (PMC11412914; doi:10.1111/jcmm.70079)
Supplement: Supplementary file 2 — Material S2. [file JCMM-28-e70079-s002.zip › 7.GSEA analysis/2.ATOH7/KEGG_NOTCH_SIGNALING_PATHWAY.html]

Details for gene set KEGG\_NOTCH\_SIGNALING\_PATHWAY[GSEA]

|  || Dataset | ATOH7.group.cls#h\_versus\_l |
| Phenotype | group.cls#h\_versus\_l |
| Upregulated in class | l |
| GeneSet | KEGG\_NOTCH\_SIGNALING\_PATHWAY |
| Enrichment Score (ES) | -0.14378792 |
| Normalized Enrichment Score (NES) | -0.54242027 |
| Nominal p-value | 0.95991564 |
| FDR q-value | 0.97688293 |
| FWER p-Value | 1.0 |
Table: GSEA Results Summary

  

Fig 1: Enrichment plot: KEGG\_NOTCH\_SIGNALING\_PATHWAY      
 Profile of the Running ES Score & Positions of GeneSet Members on the Rank Ordered List

  

| SYMBOL | TITLE | RANK IN GENE LIST | RANK METRIC SCORE | RUNNING ES | CORE ENRICHMENT || 1 | NUMBL | na | 2205 | 0.329 | 0.0121 | Yes |
| 2 | DLL1 | na | 2593 | 0.303 | 0.0707 | Yes |
| 3 | DLL3 | na | 3644 | 0.250 | 0.0979 | Yes |
| 4 | RBPJL | na | 4415 | 0.221 | 0.1265 | Yes |
| 5 | NOTCH4 | na | 7372 | 0.134 | 0.0721 | No |
| 6 | HES1 | na | 9049 | 0.098 | 0.0463 | No |
| 7 | DVL2 | na | 9335 | 0.093 | 0.0595 | No |
| 8 | LFNG | na | 9597 | 0.088 | 0.0723 | No |
| 9 | JAG1 | na | 9757 | 0.085 | 0.0873 | No |
| 10 | EP300 | na | 10138 | 0.079 | 0.0945 | No |
| 11 | ADAM17 | na | 10662 | 0.071 | 0.0958 | No |
| 12 | NUMB | na | 10670 | 0.071 | 0.1119 | No |
| 13 | APH1A | na | 11156 | 0.064 | 0.1126 | No |
| 14 | MAML2 | na | 11572 | 0.058 | 0.1140 | No |
| 15 | JAG2 | na | 11922 | 0.053 | 0.1162 | No |
| 16 | SNW1 | na | 12302 | 0.049 | 0.1165 | No |
| 17 | DVL3 | na | 12971 | 0.041 | 0.1066 | No |
| 18 | HDAC2 | na | 13042 | 0.040 | 0.1138 | No |
| 19 | DVL1 | na | 13325 | 0.037 | 0.1142 | No |
| 20 | RFNG | na | 14409 | 0.027 | 0.0891 | No |
| 21 | KAT2B | na | 14487 | 0.026 | 0.0928 | No |
| 22 | PSENEN | na | 14523 | 0.026 | 0.0977 | No |
| 23 | NOTCH2 | na | 14734 | 0.023 | 0.0970 | No |
| 24 | RBPJ | na | 15331 | 0.018 | 0.0840 | No |
| 25 | NOTCH1 | na | 15401 | 0.018 | 0.0861 | No |
| 26 | DTX4 | na | 16063 | 0.012 | 0.0699 | No |
| 27 | NCOR2 | na | 16698 | 0.007 | 0.0532 | No |
| 28 | CIR1 | na | 16898 | 0.005 | 0.0487 | No |
| 29 | MAML1 | na | 17462 | 0.000 | 0.0326 | No |
| 30 | DTX3 | na | 17595 | -0.001 | 0.0289 | No |
| 31 | DTX2 | na | 18616 | -0.010 | 0.0017 | No |
| 32 | HDAC1 | na | 19008 | -0.013 | -0.0066 | No |
| 33 | KAT2A | na | 20227 | -0.024 | -0.0362 | No |
| 34 | NCSTN | na | 20251 | -0.024 | -0.0313 | No |
| 35 | PSEN2 | na | 20477 | -0.027 | -0.0317 | No |
| 36 | CTBP2 | na | 22714 | -0.051 | -0.0844 | No |
| 37 | NOTCH3 | na | 22726 | -0.051 | -0.0729 | No |
| 38 | PSEN1 | na | 22990 | -0.055 | -0.0680 | No |
| 39 | CTBP1 | na | 24172 | -0.072 | -0.0855 | No |
| 40 | CREBBP | na | 24831 | -0.083 | -0.0855 | No |
| 41 | HES5 | na | 26855 | -0.122 | -0.1157 | No |
| 42 | DLL4 | na | 27725 | -0.144 | -0.1077 | No |
| 43 | DTX3L | na | 28516 | -0.165 | -0.0926 | No |
| 44 | PTCRA | na | 29830 | -0.205 | -0.0834 | No |
| 45 | MAML3 | na | 30720 | -0.237 | -0.0545 | No |
| 46 | DTX1 | na | 32291 | -0.312 | -0.0281 | No |
| 47 | MFNG | na | 33608 | -0.427 | 0.0323 | No |
Table: GSEA details [plain text format]

  

Fig 2: KEGG\_NOTCH\_SIGNALING\_PATHWAY      
 Blue-Pink O' Gram in the Space of the Analyzed GeneSet

  

Fig 3: KEGG\_NOTCH\_SIGNALING\_PATHWAY: Random ES distribution      
 Gene set null distribution of ES for **KEGG\_NOTCH\_SIGNALING\_PATHWAY**
